# Mental health and wellbeing in spouses of persons with dementia: the Nord-Trøndelag health study

**DOI:** 10.1186/1471-2458-14-413

**Published:** 2014-05-01

**Authors:** Helga Ask, Ellen Melbye Langballe, Jostein Holmen, Geir Selbæk, Ingvild Saltvedt, Kristian Tambs

**Affiliations:** 1 Division of Mental Health, Norwegian Institute of Public Health, P.O. Box 4404, Nydalen N-0403 Oslo, Norway; 2 Centre for Old Age Psychiatric Research, Innlandet Hospital Trust, P.O. Box 68, 2312 Ottestad, Norway; 3 Department of General Practice and Public Health, HUNT Research Centre, Norwegian University of Science and Technology (NTNU), Levanger, Norway; 4 Department of Neuroscience, Norwegian University of Science and Technology (NTNU) and Department of Geriatrics, St Olav Hospital, University Hospital of Trondheim, Trondheim, Norway

**Keywords:** Dementia, Caregiving, Partner, Depression, Anxiety, Life satisfaction, Ageing

## Abstract

**Background:**

Caring for a spouse diagnosed with dementia can be a stressful situation and can put the caregiving partner at risk of loss of mental health and wellbeing. The main aim of this study was to investigate the relationship between dementia and spousal mental health in a population-based sample of married couples older than 55 years of age. The association was investigated for individuals living together with their demented partner, as well as for individuals whose demented partner was living in an institution.

**Methods:**

Data on dementia were collected from hospitals and nursing homes in the county of Nord-Trøndelag, Norway. These data were combined with data on spousal mental health, which were collected in a population-based health screening: the Nord-Trøndelag Health Study (HUNT). Of 6,951 participating couples (>55 years), 131 included one partner that had been diagnosed with dementia.

**Results:**

Our results indicate that after adjustment for covariates, having a partner with dementia is associated with lower levels of life satisfaction and more symptoms of anxiety and depression than reported by spouses of elderly individuals without dementia. Spouses living together with a partner diagnosed with dementia experienced moderately lower levels of life satisfaction (0.35 standard deviation [SD]) and more symptoms of depression (0.38 SD) and anxiety (0.23 SD) than did their non-caregiving counterparts. Having a partner with dementia that resided in a nursing home was associated with clearly lower life satisfaction. Compared with non-caregivers, these spouses reported lower levels of life satisfaction (1.16 SD), and also more symptoms of depression (0.38 SD), and more symptoms of anxiety (0.42 SD).

**Conclusions:**

Having a partner with dementia is associated with loss of mental health and reduced life satisfaction. The risk of adverse mental health outcomes is greatest after the partner’s nursing home admission.

## Background

Dementia is a major cause of disability and suffering among older people [1]. Being a partner of an individual with a chronic, degenerative illness like dementia can be highly stressful and challenging. The literature clearly documents that caring for a person with dementia can be associated with loss of mental health and subjective wellbeing [2-15]. Studies have reported that 20-50% of dementia caregivers develop depression or high levels of depressive symptoms [4,6], and that these rates are stable or increasing over time [2,16]. A recent prospective cohort study estimated the incidence of depression among spouses of persons with dementia to be more than fourfold higher than among spouses of persons without dementia [7]. Caregivers of dementia patients also experience higher levels of depressive symptoms compared with caregivers of physically impaired older adults [17]. The manifestation of anxiety among caregivers has received less attention [18]. Some studies have reported that clinically significant anxiety affects approximately a quarter of dementia caregivers [5,8], while other studies have reported lower or no higher risk of anxiety [4,7].

Various models of stress and coping, often based on the model formulated by Pearlin and colleagues [19-21], have served as theoretical frameworks for investigating negative consequences of dementia caregiving. Caregiving is defined as a chronic stress process that places the caregiver at risk for negative outcomes. Dementia caregivers must manage functional and cognitive impairment and often encounter behavioral problems and personality changes in the people for whom they care [22]. These factors are defined as primary stressors in the model and are related to the amount of care needed. Primary stressors activate secondary stress factors associated with roles and activities outside the caregiving context (e.g., related to work or other family members and friends). Together, all of these factors generate a stressful situation that could cause a loss of mental health and wellbeing in caregivers.

A variety of contextual and personal factors may mediate and/or moderate the relationship between stressors and outcomes. One possible mediating factor is whether or not the caregivers live together with their demented spouse. Most persons with dementia are cared for at home during the early stages of the illness [23]; as the need for care grows, and perhaps as the capacity to provide appropriate care decreases with increasing age, many move to nursing homes. The fact that the caregiving spouses must live alone, and no longer can offer daily care for their demented partners, might partly explain a negative emotional outcome. Such a possible mediation effect has rarely been tested [23]. The residential situation of the demented person might also be considered as a *moderator* of the outcome. Because nursing home admission relieves the spouse of primary responsibility, it might be expected to offer some benefit for caregivers. Although some studies have suggested that such relief takes place [24-26], the majority of previous results indicate that caregiver depressive symptoms remain stable or even increase after nursing home admission [23,27-31].

Social isolation and decreased leisure time are examples of secondary stressors in the stress process model that are assumed to mediate the relationship between the dementia diagnosis and the spousal caregiver outcome. Providing care is associated with reduced time and fewer opportunities for socializing and leisure pursuit, activities associated with wellbeing [12,14]. Likewise, caregivers often have less time and energy for exercise and other health-promoting activities and may neglect their own health to provide necessary care [14]. A possible mediating effect of caregiver physical health is largely untested [32]; however, some studies indicate poor self-rated health status to be a significant indicator for depression in caregivers [33].

The extent to which the caregiving process results in loss of mental health and wellbeing is determined in part by exacerbating and mitigating factors that affect the caregiver’s appraisal of the situation [14]. Although studies report conflicting findings, research has shown that the loss of mental health and wellbeing associated with having a spouse with dementia increases with age [10,29], female sex [10,34,35], and low income [29,35,36]. Social support is considered to buffer negative effects of caregiving [21,29,35,37,38]; however, the evidence is inconsistent [5]. The literature also suggests that caregiver characteristics related to health status, religious faith, personality traits, coping styles, self-esteem, mastery, and optimism might affect the appraisal of the stressors [14,29,37,39,40].

There are several inconsistencies in the literature on mental health in caregivers of persons with dementia. Most striking is the wide variation of the prevalence estimates of poor psychological health of caregivers [11]. Further, no clear consensus exists regarding whether and how the mentioned possible mediating or moderating variables in fact affect the outcomes [14,41]. Literature reviews have concluded that methodological and conceptual limitations can explain much of this variation and inconsistency [13,14,42-44].

One reason for the divergent results might be the composition of the caregiver samples. The majority of previous studies do not differentiate between spouses, adult children, and other relatives as caregivers, and much of the research has been on the female caregiver of the person with dementia, with little attention given to the male caregiver [34]. Only a small number of studies have compared homogeneous samples of dementia caregivers and non-caregivers [10]. Most studies have relied on relatively small convenience samples with a high risk of over-representing highly distressed caregivers. The lack of control groups make it impossible to adjust for possible confounders [14,42], and inadequate covariate coverage may in turn lead to inflated associations of the unique stressors of caregiving [11,14].

Only a few studies e.g. [7,45] have used large population-based samples to investigate the emotional costs for caregivers, by comparing them to the non-caregiving population [14]. An exception is a study by Joling et al. (2010) that employ an unselected nationally representative population [7]. However, like most studies that only involve spousal caregivers, this study only consider at-home caregivers. Most individuals with dementia move into nursing homes once their dementia progresses to a certain stage. Therefore, it is also crucial to explore the possible loss of mental health associated with having a demented spouse outside of the home.

Using a large population-based sample, the main aim of our study was to investigate the possible loss of mental health and wellbeing for individuals living with their demented partner and for individuals that have a demented partner in a nursing home, by comparing them to spouses of elderly people without dementia. Our second goal was to investigate to what extent variables such as subjective health, leisure time activities, and social support mediates the relationship between having a partner with dementia and caregiver outcomes. Our third goal was to investigate the effect of personal and environmental variables possibly moderating the relationship between having a partner with dementia and caregiver outcomes. With a relatively large sample, we could also examine possible sex differences in the outcomes.

## Methods

This study is based on data from three sources: the Health and Memory Study (HMS), from which the dementia diagnoses were obtained; the Nord-Trøndelag Health Study (HUNT), which contains data on mental health and wellbeing; and public registry data made available from the governmental statistics agency Statistics Norway (SN), which includes demographic and kinship information. Both the HMS and the HUNT study were conducted in the county of Nord-Trøndelag (NT) in Norway. The NT population consists of around 94,000 adults and is fairly representative of the general Norwegian population in terms of geography, economy, industry, and age distribution [46]. The three data materials were matched on the basis of the national identification number assigned to all Norwegian citizens, which was included in all three files. This number was removed for personal privacy concerns before the matched dataset was made available to the researchers. The study was approved by the Regional Committee for Medical and Health Research Ethics (REC) and was conducted in accordance with the rules of the Helsinki declaration.

Data for HMS were collected through two procedures for details, see [47]. First, during the period 2008–2010, anamnestic data from 1998–2010 in the two hospitals in NT were examined to find patients that had been registered with a dementia diagnosis. Second, during 2010–2011, all inhabitants residing in nursing homes in NT were invited to participate in an extensive health examination focused on dementia diagnoses and related variables, using standardized interviews to assess cognitive decline and dementia. A total of 1332 dementia cases were identified: 104 were identified in both hospital and nursing home data, 727 were registered from hospital files, and 501 were registered from nursing homes.

To identify the spouses of persons with dementia, as well as married couples in the rest of the NT population, the diagnostic information was matched with data from SN containing information about who was married to whom during the study period.

To obtain information about mental health and wellbeing, we matched HMS and SN data with data collected for HUNT3 during the period from 2006 to 2008 [48]. The entire NT population aged 20 years or older was invited to participate. Data from two HUNT3 questionnaires (Q1 and Q2) were included in our analyses. Q1 was sent together with the invitation and returned at the screening site by 50,807 of the invited citizens (54%). Q2 was handed out at the screening stations and returned by prepaid mail by 81% of the Q1 respondents. In most cases, Q2 was returned only a few days after the examination.

### Sample

**Figure 1:**
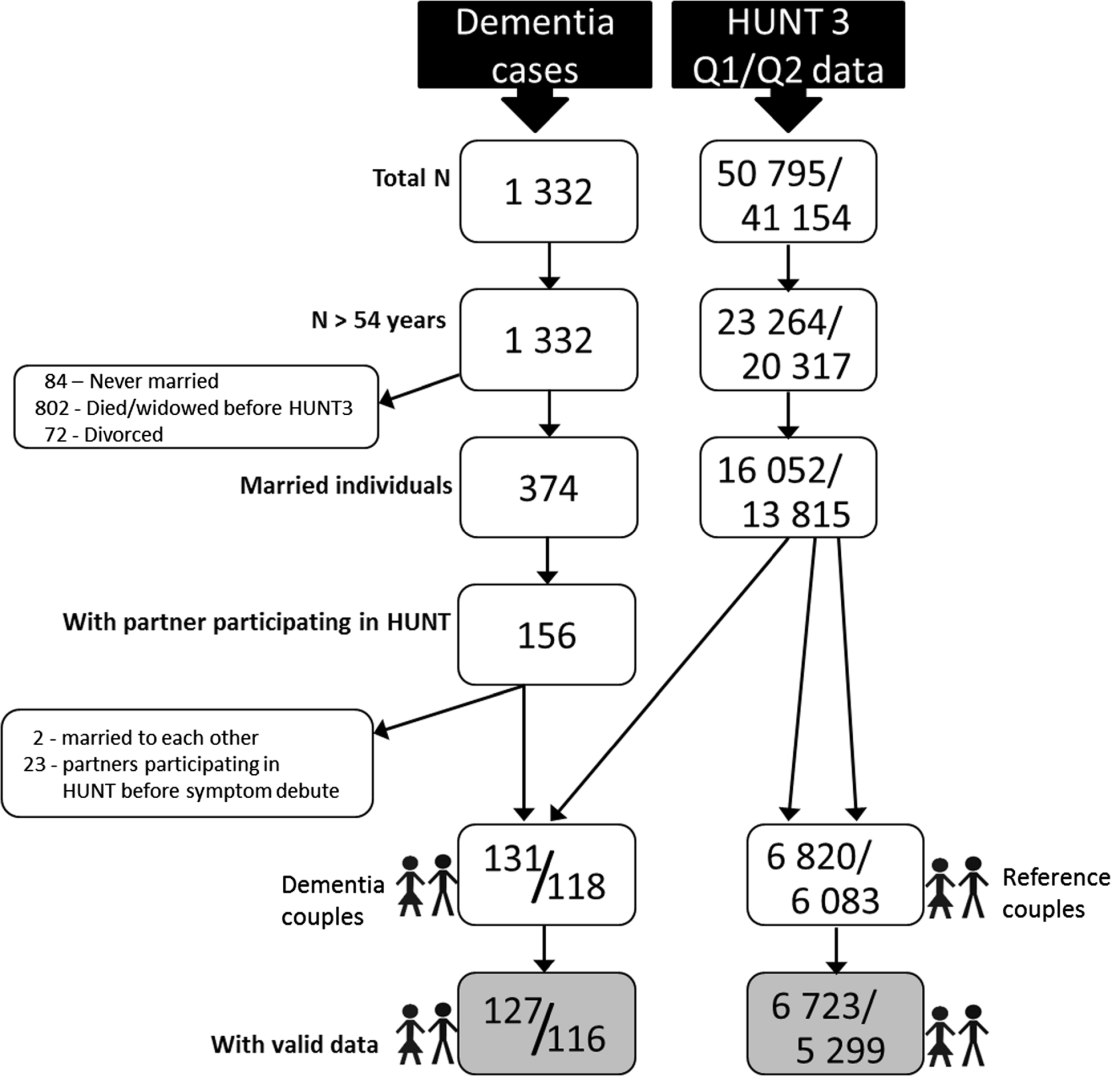
Visualizing the composition of the study sample.

The composition of the study sample is visualized in Figure 1. In HUNT3, 45.8% of the Q1 participants and 49.4% of the Q2 participants were over 54 years of age. In this age group, 68.4% of the participants were registered as married or cohabiting at the time the examination took place. Mean age was 66.4 years (range = 56–98, SD [standard deviation] = 7.7), and the group consisted of 51.5% men.

Of the 1332 persons with dementia, 374 were registered as married at the time of the HUNT3 collection. Of these, 156 had a partner participating in Q1. Two of the dementia cases were married to each other and were excluded from the sample. In addition, 23 of the partners participated in HUNT before the registered onset of dementia symptoms and were therefore excluded, leaving 131 complete couples in which one partner had a dementia diagnosis and the other partner had completed Q1. Of these, a total of 118 spouses responded to Q2. The dementia caregivers were compared with the rest of the couples in NT that were over 55 years of age. There were 6,820 couples in which the caregiver reference had Q1 data and 6,083 with Q2 data.

Only respondents with valid data on all independent and outcome variables from Q2 (after treatment of missing values) were included in the sample, resulting in 116 caregivers and 5,299 reference couples. However, for the purpose of one analysis, the total Q1 sample with valid data on life satisfaction was included: 127 dementia caregivers and 6,723 reference couples.

As part of preparing the data file for analyses, data on partners was first arranged as a double entry file; each individual was included both as an index person on one record and as the spouse of the index person on another, mirrored record. In couples with a spouse with dementia, the record with the dementia case as the index person was retained and the mirrored record was deleted. Each couple without dementia was included only once; partners were randomly assigned to be either an index person or a spouse.

### Measures

#### *Outcome measures*

Of the 6,951 Q1 participants, 98.5% responded to one item measuring life satisfaction. The item was phrased as follows: “Thinking about your life at the moment, would you say that you by and large are satisfied with life, or are you mostly dissatisfied?” The seven response categories ranged from “extremely satisfied” to “extremely dissatisfied”, scored 1–7; a higher score indicated increased dissatisfaction. Other studies have shown that reported responses to such questions are quite valid and fairly reliable [49]. The score was standardized and used as a continuous outcome measure.

The Hospital Anxiety and Depression Scale (HADS) [50], an established instrument used to assess symptoms of anxiety and depression [51], was included in Q2. The scale consists of 14 four-point Likert-scaled items, seven for anxiety (e.g. “I can sit at ease and feel relaxed”) and seven for depression (e.g. “I feel as if I’m slowed down”). The seven-item CONOR Mental Distress Index (CMD), with three items assessing anxiety and four assessing depression, was included in Q1. CMD is described in detail elsewhere [52]. Depression items on negative affectivity are missing in the HADS; therefore, to include such symptoms, we combined the HADS and CMD into a single instrument with 11 depression symptoms and 10 anxiety symptoms. Cronbach’s alphas were 0.80 and 0.85 for the depression and anxiety scales, respectively. Before they were standardized and used as continuous outcome measures, the scores were logarithmically transformed to approximate a normal distribution.

#### *Main predictor*

Our principal predictor was the presence or absence of a dementia diagnosis. The 10^th^ revision of the International Classification of Diseases (ICD-10) was used to set the dementia diagnoses and to classify the cases as arising from Alzheimer’s disease (AD), vascular dementia (VaD), mixed AD/VaD, frontotemporal dementia, dementia with Lewy body, and other dementias (for details, see 47).

#### *Potential mediators and moderators*

The respondents reported in Q2 whether they were living alone or living with a spouse/partner (‘yes’ or ‘no’). The two items were combined into one dichotomous variable in which ‘0’ referred to living together with a spouse and ‘1’ referred to living alone. Regarding the dementia case group, we assumed that living alone indicated that the spouse with dementia was living in a nursing home. The variable was supplemented with a variable from the HMS data containing the nursing home admission date. If this date preceded the date for the partner’s participation in HUNT, the spousal caregiver was coded as ‘living alone’.

Subjective health was measured by one item in Q1, “How is your health at the moment?”, with four response alternatives ranging from ‘poor’ (0) to ‘very good’ (3). The use of one-item measures of subjective health is supported in the literature [53]. The sum of four additional items in Q1 pertained to the extent to which functioning in daily life was impaired by longstanding (at least 1 year) physical illness or injury regarding motor ability, vision, hearing, or physical illness. Response categories ranged from ‘not impaired’ (0) to ‘severe’ (3).

Receptive cultural activities were assessed in Q2 by four separate items phrased as follows: “How often in the last 6 months have you been to a museum or art exhibition, a concert/theater/film, a church/chapel, sport events?” Response alternatives ranged from ‘never’ (0), to ‘more than three times a month’ (3). Creative cultural activities were assessed in Q2 by six separate items: “How many times in the last 6 months have you participated in the following: an association activity or club meeting, music/singing/theater, parish work, outdoor activities, dance, and exercise/sports?” The response alternatives ranged from ‘never’ (0), to ‘more than once a week’ (4). Indices for the receptive and the creative cultural activities were computed by summing the item scores. These measures have been used in previous publications [54].

Social support was measured by the sum of two items in Q2, which were phrased as follows: “Do you have friends who can help you when you need them?” and “Do you have friends whom you can speak to in confidence?” The responses were ‘yes’ (1) or ‘no’ (0).

Q2 included a short version of the Eysenck Personality Questionnaire measuring extraversion [55], shown to correlate 0.90 with the total scale [56]. Respondents were asked to describe themselves as they normally are, answering yes or no to six statements. A copy of all Q2 items is available at http://tinyurl.com/HUNT3Q2.

Two items in Q2 were related to view of life. The first item, “Which life philosophy is most like yours?” with response categories of ‘Christian’, ‘Humanistic’, ‘Atheistic’, or ‘Other’, coded ‘1’ for Christian and ‘0’ for the other alternatives. The second item was derived from the positive sub-scale of the religious coping instrument RCOPE [57]. It was phrased as follows: “I seek God’s help when I need strength and solace”, with the response categories ‘never’, ‘sometimes’, and ‘often’, scored 0–2. The two items were standardized and summed. Another coping-related item was included in Q2, phrased, “When something bad happens in my life, I think that it happened for a purpose”, with responses ‘no’, ‘don’t know’, and ‘yes’, scored 0, 0.5, and 1. A detailed description of the three items is provided elsewhere [58].

Sex, age, education (scored 1–5), income, number of children, age difference between spouses, and place of residence (living in one of the four towns or in the more rural areas of the county) are possible confounders, and are also moderators of the relationship between the caregiver burden (spousal dementia) and negative caregiver outcomes. These variables were obtained from the SN registry data. Because preliminary analysis suggested that age had a U-shaped relationship with outcome, age was included as a categorical variable coded with 5-year intervals.

### Treatment of missing values

In cases in which the respondent had valid data for at least one-half of the items of a scale, we used the SPSS Missing Value Analyses (MVA), Expectation Maximization (EM), and allowed items with valid data within the same instrument or set of items to predict values replacing missing values. At least one missing value was replaced in 14.4% of the Q2 sample for symptoms of anxiety and depression, in 7.2% of the sample for extraversion, and in 1.7% of the respondents for religion.

Of the Q2 sample with valid data on the mental health outcome variables (N = 6,083), 15 participants had unknown or missing education. These were coded with the lowest educational level (1) and included in the analyses. A total of 122 participants did not report any functional impairment; their missing values were replaced by 0, indicating no impairment.

After this missing value treatment, the amount of missing values in the dementia case group ranged from 1.7% to 9.5% in the possible confounding, mediating, or moderating variables. To avoid shrinkage of the group, we chose to take some additional steps. New values on subjective health were predicted from earlier reported subjective health in HUNT2 and/or HUNT1 for 5 caregivers, using EM. Receptive cultural activities and/or creative cultural activity were scored with the median values, 2 and 4, for 8 and 11 caregivers with missing values, respectively. For caregivers who had still missing values on extraversion (N = 5), religion (N = 6), or “happens for a purpose” (N = 6), new values were predicted (in separate EM analyses) from 25 other HUNT variables containing questions about lifestyle, memory, neighborhood, and occupation. Regressing these 25 variables on extraversion, religion, and “happens for a purpose” gave multiple R-values of 0.62, 0.78, and 0.81, respectively.

### Statistical analysis

Multivariate ANOVA (SPSS General Linear Models, Unianova) was conducted separately for each of the three outcome measures (life satisfaction, depression, anxiety). Dementia diagnosis was entered as the main predictor (stressor) for spousal loss of wellbeing and mental health. The variable contained three values: no dementia (0), dementia and living at home (1), and dementia and living in nursing home (2). First, unadjusted associations between the two dementia groups and negative caregiver outcomes were observed. Next, confounding variables were included stepwise, adding one new predictor variable at a time. Caregiver age was entered on the first step, followed by sex, education, couple income, and age difference between spouses.

Each variable that was classified as a possible mediator according to the stress process model [20,21] was entered in separate analyses together with the confounding variables. This approach allowed us to track the change that each mediator variable elicited in the model. Three types of mediator variables were entered: own physical health (two variables: subjective health and impairment of daily functioning), cultural activities (two variables: receptive and creative activity), and social support. Ultimately, all variables were entered into the model simultaneously, which provided estimates of the unique direct association between caregiver outcomes and each predictor.

Possible moderating effects of extraversion, religion, meaning, number of children, and place of residence were tested, together with the 11 possible interaction terms, between the dementia diagnosis and the confounders and mediators described above. A Bonferroni correction of the alpha level 0.05 (β = 0.05/16) gave an alpha level of 0.003.

Finally, we ran one analysis that employed a dichotomous dementia predictor with values 0 (no dementia) and 1 (dementia). Aside from including living arrangement (home versus nursing home) as a mediator variable, all confounding and mediator variables were identical to those in the previous analyses.

Because the outcome variables were standardized, results are reported as adjusted mean group scores above or below the reference group in fractions of standard deviations.

## Results

### Descriptive statistics

The mean age of the caregiving spouses was 74.0 years old (SD = 7.7, range: 56–92 years). The mean age of the reference spouses was 66.5 years (SD = 7.2, range: 56–93 years). Table 1 presents demographic descriptive statistics for the caregivers and the reference spouses. Mean age was 74.8 years in the dementia case group (SD = 7.4, range: 57–92 years) and 66.5 years in the non-dementia group (SD = 7.2, range: 56–92 years). Of the dementia cases included in our sample, diagnoses were as follows: 44.8% with AD, 12.1% with VaD, 12.1% with mixed AD/VaD, 11.2% with frontotemporal dementia, 5.2% with dementia with Lewy body, and 8.6% with other dementias.

**Table 1:** Descriptive statistics

	***Caregiving spouses N*** = ***116***	***Q2***-***sample N*** = ***5 299***
	**N (% of N)**	**N (% of N)**
***Gender***		
Men	50 (56.9)	2621 (49.5)
Women	66 (43.1)	2678 (50.5)
***Age***		
> 85 years	6 (5.2)	37 (0.7)
81-85 years	19 (16.4)	199 (3.8)
76-80 years	28 (24.1)	454 (8.6)
71-75 years	24 (20.7)	779 (14.7)
66-70 years	25 (21.6)	1135 (21.4)
61-65 years	7 (6.0)	1463 (27.6)
56-60 years	7 (6.0)	1232 (23.2)
***Education***		
1-7 years	28 (24.1)	638 (12.0)
7-10 years	9 (7.8)	655 (12.4)
11-13 years	49 (42.2)	2284 (43.1)
14 years	12 (10.3)	664 (12.5)
> 14 years	18 (15.5)	1058 (20.0)
***Urban versus rural***		
Rural areas	49 (42.2)	1905 (36.0)
Town 5	11 (9.5)	407 (7.7)
Town 4	10 (8.6)	517 (9.8)
Town 3	15 (12.9)	657 (12.4)
Town 2	17 (14.7)	1018 (19.2)
Town 1	14 (12.1)	795 (15.0)
***Living arrangements***		
Living alone	30 (25.9)	26 (0.5)
Living with spouse	86 (74.1)	5273 (99.5)

### Negative caregiver outcomes

Table 2 depicts the unadjusted and adjusted effects of having a partner with dementia on life-satisfaction and depressive and anxiety symptoms. The unadjusted effects indicate that dementia caregivers with a partner in a nursing home score one SD poorer on life satisfaction (*B* = 1.07), while persons living together with their demented partner score one-fourth of an SD (*B* = 0.28) lower on life satisfaction compared with the rest of the population. As indicated by the far from overlapping confidence intervals (CI) for these estimates, the differences between the two dementia caregiver groups is highly significant (*t* = 4.87, *p* < 1 × 10^-6^). After adjustment for the potentially confounding effect of demographic variables (age, sex, income, education, and spousal age difference), the group differences for life satisfaction increased to 1.16 SD (Δ*B* = 0.09) for nursing home caregivers and 0.35 (Δ*B* = 0.07) for the at-home caregivers. The estimates for the difference between the two dementia groups remained highly significant (*p* < 1 × 10^-6^).

**Table 2:** **Crude and adjusted associations between dementia diagnosis and partners**’ **outcomes**

	**Life satisfaction**					**Depressive symptoms**							**Anxiety symptoms**					
	**Spouse at institution**			**Living together**		**Spouse at institution**				**Living together**			**Spouse at institution**			**Living together**		
	***B***	***CI***	***p***	***B***	***CI***	***p***	***B***	***CI***	***p***	***B***	***CI***	***p***	***B***	***CI***	***p***	***B***	***CI***	***p***
**0**	1.07	0.71 – 1.44	***	0.28	0.06 – 0.49	*	0.52	0.16 – 0.89	*	0.48	0.27 – 0.69	***	0.46	0.10 – 0.83	*	0.27	0.06 – 0.49	**
***Adjusted for confounders***																		
**1**	1.07	0.71 – 1.44	***	0.27	0.06 – 0.49	*	0.52	0.16 – 0.89	*	0.49	0.28 – 0.70	***	0.46	0.10 – 0.82	*	0.25	0.04 – 0.46	*
**2**	1.16	0.80 – 1.53	***	0.35	0.14 – 0.57	**	0.40	0.03 – 0.76	*	0.38	0.17 – 0.59	***	0.43	0.02 – 0.44	*	0.23	0.02 – 0.44	*
**3**	1.16	0.80 – 1.52	***	0.34	0.13 – 0.56	**	0.38	0.02 – 0.74	*	0.38	0.16 – 0.59	***	0.42	0.06 – 0.78	*	0.23	0.02 – 0.44	*
**4**	1.16	0.79 – 1.52	***	0.35	0.14 – 0.57	**	0.38	0.02 – 0.74	*	0.38	0.17 – 0.60	***	0.42	0.06 – 0.79	*	0.23	0.02 – 0.44	*
***Adjusted for all confounders and each of the mediators***																		
**5**	1.09	0.75 – 1.43	***	0.30	0.10 – 0.50	**	0.32	-0.02 – 0.66		0.34	0.14 – 0.54	***	0.37	0.03 – 0.72	*	0.19	-0.01 –0.39	
**6**	1.13	0.77 – 1.49	***	0.33	0.12 – 0.55	**	0.35	-0.01 – 0.71		0.36	0.15 – 0.57	***	0.41	0.05 – 0.77	*	0.22	0.01 – 0.43	*
**7**	1.14	0.78 – 1.49	***	0.35	0.14 – 0.56	**	0.35	0.00 – 0.70		0.38	0.18 – 0.59	***	0.41	0.05 – 0.77	*	0.23	0.02 – 0.44	*
***Adjusted for all confounders and all mediators***																		
	1.06	0.73 – 1.40	***	0.28	0.14 - 0.50	**	0.28	-0.05 – 0.62		0.33	0.13 - 0.52	***	0.36	0.01 – 0.70	*	0.19	-0.01 -0.39	

0 = Unadjusted; 1 = Gender; 2 = 1 + Age; 3 = 2 + Income and education; 4 = 3 + Spousal age difference; 5 = 4+ Subjective health/impaired functioning; 6 = 4 + cultural activities; 7 = 4 + social support.

***p < 0.001 **p < 0.01 *p < 0.05. B-values indicate differences between the dementia caregivers and the reference spouses in fractions of standard deviation.

The unadjusted estimates indicate that dementia caregivers report more symptoms of depression than non-caregivers, whether their partner resides in an institution (0.52 SD) or at home (0.48 SD). After adjustment for the demographic variables, the *B*-values decreased to 0.38 SD for both groups (Δ*B* = -0.14 and -0.10).

According to the unadjusted effects data, dementia caregivers with spouses at nursing homes scored 0.46 SD higher on anxiety symptoms than did the reference group; at-home caregivers scored 0.27 SD higher. Adjusted effects were 0.42 (Δ*B* = -0.04) and 0.23 (Δ*B* = -0.04), respectively. The difference between the two caregiver groups was not statistically significant.

### Mediating effects

Potentially mediating variables were included in separate analyses adjusted for demographic variables. Adjusting for own physical health reduced the *B* estimates by 0.07 and 0.05 for life satisfaction, 0.06 and 0.05 for depressive symptoms, and 0.05 and 0.04 for anxiety. The observed reduction in fractions of SD when including cultural activities and social support were minor (all changes ≤ 0.03).

When all potential confounders and mediators in the model were included simultaneously (the last line in Table 2), the effects on both caregiver groups remained significant for all outcomes except for depression in nursing home caregivers and anxiety symptoms reported by at-home caregivers. Compared with the reference group, nursing home caregivers scored 1.06 SD lower on life satisfaction and 0.36 SD higher on symptoms of anxiety, while at-home caregivers scored 0.28 SD lower on life satisfaction and 0.33 SD higher on symptoms of depression.

Table 3 presents crude and fully adjusted main effects of all independent variables on the outcome measures when we employed a dichotomous dementia predictor and included living arrangement as a mediator. The first two analytic steps of this analysis were estimating the unadjusted effect and adjusting for demographic variables. With life satisfaction as outcome, these first two steps included only variables from Q1, which was completed by a somewhat larger sample than what completed Q2. To determine whether attrition between Q1 and Q2 might have affected the results, we ran the first two steps of the analysis for the outcome ‘life satisfaction’ in the total Q1 sample (increasing the number of dementia cases from 113 to 127). The results indicated no large differences between the two samples; the unadjusted effect changed from 0.48 to 0.47, and the adjusted effect changed from 0.49 to 0.51 (not reported in tables).

**Table 3:** Crude and fully adjusted main effects of the predictor variables on spousal mental health and life satisfaction

	**Life satisfaction**		**Symptoms of depression**		**Symptoms of anxiety**	
	**Crude *B *(*CI*)**	**Adjusted *B *(*CI*)**	**Crude *B *(*CI*)**	**Adjusted *B *(*CI *)**	**Crude *B *(*CI *)**	**Adjusted *B *(*CI *)**
Dementia Non-dementia	0.48 (0.29 – 0.67) ***0^a^	0.32 (0.14 – 0.50) ***0^a^	0.49 (0.31– 0.68) ***0^a^	0.30 (0.12 – 0.48) 0^a^	0.32 (0.14 – 0.51) *0^a^	0.21 (0.02 – 0.39) 0^a^
***Gender***				0.08 (0.03 – 0.13)**		
Men	-0.04 (-0.09 – 0.02)	0.02 (-0.03 – 0.08)	0.07 (0.02 – 0.13)**	0^a^	-0.30 (-0.36 – -0.25)***	-0.28 (-0.33 – -0.23)***
Women	0^a^	0^a^	0^a^		0^a^	0^a^
***Age***	***	***	***	-0.02 (-0.31 – 0.26)	**	**
> 85 years	-0.31 (-0.62 – -0.01)*	-0.79 (-1.08 – -0.51)***	0.52 (0.22 – 0.82)***	0.06 (-0.07 – 0.20)	-0.13 (-0.44 – 0.07)	-0.34 (-0.64 – -0.05)*
81-85 years	-0.19 (-0.34– -0.05)**	-0.48 (-0.61 – -0.34)***	0.41 (0.39 – 0.58)***	0.02 (-0.09 – 0.12)	0.04 (-0.10 – 0.10)	-0.14 (-0.29 – -0.00)*
76-80 years	-0.22 (-0.33 – -0.12)***	-0.41 (-0.51 – -0.31)***	0.28 (0.17 – 0.38)***	0.04 (-0.04 – 0.13)	0.03 (-0.14 – 0.08)	-0.13 (-0.24 – -0.02)*
71-75 years	-0.26 (-0.35 – -0.17)***	-0.35 (-0.44 – - 0.26)***	0.19 (0.10 – 0.28)***	-0.05 (-0.13 – 0.02)	-0.09 (-0.18 – 0.00)*	-0.11 (-0.20 – -0.02)*
66-70 years	-0.23 (-0.31 – -0.15)***	-0.27 (-0.35 – -0.20)***	0.03 (-0.05 – 0.11)	-0.01 (-0.08 – 0.06)	-0.15 (-0.23 – -0.07)***	-0.14 (-0.22 – -0.06)***
61-65 years	-0.11 (-0.18 – -0.03)**	-0.12 (-0.19 – -0.05)***	0.03 (-0.05 – 0.10)	0^a^	-0.12 (-0.20 – -0.05)**	-0.10 (-0.18 – -0.03)**
56-60 years	0^a^	0^a^	0^a^		0^a^	0^a^
***Income***^**b**^	-0.02 (-0.05 – 0.01)	0.00 (-0.03 – 0.03)	-0.12 (-0.15 – -0.09)***	0.00 (-0.03 – 0.03)	-0.06 (-0.09 – -0.03)***	0.01 (-0.02 – 0.04)
***Education***^**b**^	0.00 (-0.03 – 0.03)	0.06 (0.03 – 0.09)***	-0.13 (-0.15 – -0.10)***	-0.02 (-0.05 – 0.01)	-0.09 (-0.12 – -0.06)***	-0.04 (-0.07 – -0.01)*
***Spousal age difference***	-0.01 (-0.04 – 0.02)	-0.03 (-0.05 – 0.00)	-0.06 (-0.09 – -0.04)***	-0.03 (-0.05 – 0.02)	-0.01 (-0.03 – 0.02)	0.01 (-0.02 – 0.04)
***Living arrangements***				0.05 (-0.23 – 0.33)		
Living alone	0.78 (0.51 – 1.04)***	0.66 (0.40 – 0.92)***	0.35 (0.08 – 0.61)*	0^a^	0.25 (0.02 – 0.52)***	0.10 (0.17 – 0.37)*
Living with spouse	0^a^	0^a^	0^a^		0^a^	0^a^
***Subjective health***^**bc**^	0.36 (0.34 – 0.39)***	0.32 (0.29 – 0.35)***	0.37 (0.34 – 0.39)***	0.32 (0.29 – 0.35)***	0.30 (0.28 – 0.33)***	0.25 (0.22 – 0.28)***
***Impaired functioning***^**b**^	0.22 (0.19 – 0.25)***	0.09 (0.06 – 0.12)***	0.25 (0.23 – 0.28)***	0.08 (0.05 – 0.11)***	0.18 (0.15 – 0.20)***	0.07 (0.04 – 0.09)***
***Receptive cultural act***^**b**^	-0.08 (-0.11 – -0.06)***	-0.03 (-0.05 – 0.00)	-0.13 (-0.15 – -0.10)***	-0.05 (-0.08 – -0.02)***	-0.05 (-0.08 – -0.03)***	0.01 (-0.02 – 0.04)
***Creative cultural act***^**b**^	-0.08 (-0.10 – -0.05)***	-0.02 (-0.05 – 0.01)	-0.17(-0.19 – -0.14)***	-0.11 (-0.14 – -0.08)***	-0.09 (-0.11 – -0.06)***	-0.02 (-0.05 – 0.01)
***Social support***^**bc**^	0.17 (0.14 – 0.19)***	0.13 (0.10 – 0.15)***	0.23 (0.20 – 0.25)***	0.22 (0.20 – 0.25)***	0.14 (0.11– 0.16)***	0.11 (0.09 – 0.14)***

***p < 0.001 **p < 0.01 *p < 0.05 ^**a**^Reference group ^**b**^the scale is standardized ^**c**^the scale is reversed B-values indicate fractions of standard deviation.

### Moderator effects

Possible moderator effects of extraversion, religion, meaning, number of children, and place of residence (urban or rural) on the relationship between dementia and caregiver outcomes were tested by including interaction terms in the analyses. Interaction terms between the dementia diagnosis and the confounding and mediating variables were also included. A total of 16 interaction terms were tested. We observed no interaction effects that were statistically significant at the 0.01 level between dementia diagnosis and the variables mentioned above regarding any of the caregiver outcomes.

## Discussion

Using a large population-based sample of spouses of elderly people with and without dementia, accounting for important demographic variables, we had a unique possibility to obtain less biased estimates than much previous research. Our results indicate that the presence of a partner with dementia is associated with clearly lower levels of life satisfaction and somewhat more symptoms of anxiety and depression than reported by spouses of elderly persons without dementia. This association was present at the crude level, as well as after adjusting for demographic variables. Our results show that having a partner with dementia residing in a nursing home is associated with lower life satisfaction than having a partner with dementia that resides at home.

The estimated differences in our study are comparable to effect sizes presented in a large meta-analysis by Pinquart and Sörensen [10]; our crude estimated difference in depressive symptoms (0.49 SD) has overlapping CIs with an estimated mean difference of 0.58 SD, based on 81 earlier reported effect sizes. Likewise, our crude estimate for life satisfaction (0.48) lies within the CI, for a mean difference of 0.40 (based on 48 reported effect sizes). However, as with most previous single studies [43], mean estimates in the above-mentioned meta-analysis were based on heterogeneous groups of caregivers, lumping together spouses, adult children, other relatives, neighbors, and friends, as well as caregivers for people with disabilities other than dementia. Our sample included only spouses and only dementia caregivers, both identified as high-risk subgroups of caregivers [1,10,14]. Based on a smaller number of studies, mean differences between non-caregivers and only spousal dementia caregivers, yielded larger differences in depression [10] than suggested by our results. The supposedly high representativeness of our sample may explain the relatively moderate associations that we observed. The differences between caregivers and non-caregivers tend to lessen as the representativeness of a caregiver sample increases [10]. This has also been observed in previous studies that employ diagnostic information. The incidence of depression in a non-selected sample [7] was more than four-fold higher in caregivers than in non-caregivers; this finding is lower than estimates of 50%, which were based on samples recruited from psychiatric services and memory clinics [6]. The majority of studies in the dementia caregiving literature rely on self-selected distressed caregivers, which suggests that the associations observed in these samples may somewhat exaggerate the prevalence of depression among the caregiver population [44]. To some extent, our rather low estimates might also reflect the quite well organized and extensive public health service in Norway, which includes home-based care facilities for dementia patients that may relieve some of the adverse effects among spousal caregivers.

Joling et al. [7] did not find a significantly elevated risk of anxiety among their at-home caregivers [7]. An indication of a somewhat higher risk of depression than anxiety in at-home caregivers was observed in our results as well, with an adjusted differences between caregivers and the remaining population of 0.38 SD for depression and 0.23 SD for anxiety, albeit with highly overlapping CIs.

One important limitation in our study that might also account for lower main effects [44] is that we defined caregiving based solely on being a spouse of a person with dementia. We did not directly assess whether the respondents actually provided care or the amount of care provided. Our respondent population most likely included some partners that provide low levels of support, as the primary caregiving responsibility may lie in the hands of adult children or other relatives. Schulz and colleagues [44] have estimated that approximately 80% of individuals living with a disabled spouse provide care. However, as long as spouses are alive and available, older adults with dementia often receive assistance from a spouse while children may contribute assistance when a spouse is not present [37]. Nevertheless, independent of the amount of care provided, the emotional loss of a loved one may be the largest stressor for most spouses.

Our results regarding life satisfaction and anxiety symptoms indicate that caregivers that have partners in nursing homes experience a greater loss of mental health and wellbeing than at-home caregivers. This is in line with earlier studies that demonstrate increased symptoms after nursing home admission [23,27-31]. This difference might be explained by a mediating effect of living arrangement. Irrespective of caregiving status, living alone is associated with higher levels of mental distress and lower life satisfaction [60-63]. Because most people with dementia are eventually admitted to nursing homes, it is important to acknowledge that this transition does not appear to relieve any of the stress experienced by the caregivers, rather to the contrary.

Considering that life satisfaction and depression are closely related concepts, it is notable that our results reveal a significant difference in life satisfaction owing to living arrangement, but no such significant difference in symptom level of depression. Our results indicate that when the spouse stops residing at home, the value of life feels severely reduced; however, it does not usually result in mental illness.

Our analyses revealed no moderating effect of sex, counter to the common assumption in much of the caregiving literature that female caregivers report higher levels of psychological distress and lower levels of life satisfaction than male caregivers [37]. In their meta-analysis of sex-based differences, which included more than 200 studies, Pinquart and Sörensen suggested that sex-based differences were small to very small [34]; such differences may therefore have been undetectable in our study because of a lack of statistical power. Another possibility is that sex-based differences are more evident in samples of non-spousal caregivers. Spouses have less choice in selecting specific caregiving tasks, and (unlike sons and daughters) husbands and wives might experience the situation fairly similarly [34].

### Methodological considerations

One advantage in our study is that the self-reported measures of life satisfaction and mental health were obtained without participant knowledge of the specific research aims of the dementia study. Few other studies of caregiver mental health have data reported under such contextual neutrality as does HUNT. Being aware of the purpose of the study would most likely result in over-reporting of symptoms and loss of wellbeing because respondents would be mentally directed at focusing on problems concerning partner illness. On the other hand, our effect sizes may also have been artificially reduced, because the short scales that we used to measure psychological constructs may provide less valid responses than might be provided by more comprehensive measures.

A second advantage of our study is that there was initially no selection bias of elderly persons with or without dementia and their partners, because participants were obtained from the population aged over 55 years and living in Nord Trøndelag. However, we cannot completely rule out the possibility of a selection in the HUNT study, in which people with the most severe caregiver burden have been less likely than the remaining population to participate. Only 48.8% of registered spouses of dementia cases responded to Q1, and 42.8% responded to Q2. It is possible that the spouses with the heaviest caregiver burden chose not to participate in the HUNT study. People that are struggling with severe problems tend to be underrepresented in population surveys [63].

In addition, not all dementia cases in the county of NT were registered in our sample. Several individuals with dementia live with the disease for several years before diagnosis. However, even if a relatively large number of false negatives are present, they constitute only a small fraction of the large group of non-cases and are not critical for the estimated group differences.

In earlier studies using control groups, caregivers of dementia patients were compared either with non-caregivers or with caregivers for individuals with other types of disabilities. However, because our reference sample of spouses constitutes the general population, in addition to the presence of unregistered dementia caregivers, this group will also include caregivers for all other types of disabilities in spouses, children, and other relatives. This inclusion may have deflated our estimated group differences.

One limitation is related to the difference in life satisfaction between spouses of institutionalized demented persons and at-home caregivers. The difference may well imply that the loss feels much stronger when the demented spouse is no longer present at home; it may also reflect a negative course of dementia. Most individuals with dementia stay at home for as long as possible; they are only moved to a nursing home when the dementia becomes so severe that living at home is no longer an option. Our data cannot determine to what extent the lower life satisfaction among spouses of individuals with dementia residing in nursing homes were caused by progressive dementia illness.

Our study is limited by the fact that it is based on cross-sectional data, a design that by definition does not permit causal conclusions. However, while we cannot empirically decide whether dementia diagnosis affects spousal mental health or spousal mental health affects dementia diagnosis in the partner, the former is undoubtedly more likely than the latter.

## Conclusions

Our results indicate that having a partner with dementia is associated with loss of mental health and wellbeing, especially when the demented partner lives in a nursing home. Based on our broad definition of caregiving and the possible under-representativeness of the most severe caregiver burdens in the HUNT study, our estimates might well represent underestimations of the actual negative outcomes of spousal caregiving.

Still the moderate effect sizes in this study do not indicate that a large proportion of caregivers reach a symptom level of anxiety and depression that reflects clinical mental disorder. When symptom counts is the only measurement assessments method used it is difficult to evaluate the clinical significance of the results. However, our findings are not without practical value. With the ageing of the population, the total number of people with dementia is projected to nearly double in the next 20 years [1], as will the number of partners that enter the role of caregiver. This projected increase represents a global challenge. Knowledge that can help target specific interventions and support programs to those who need them the most is required, and may benefit both individuals and society. For example, targeting subgroups of individuals that experience high distress symptoms might be a particularly effective strategy. Our study expands the current understanding of the relationship between dementia diagnosis and spousal outcomes, addressing both mediating and moderating variables. However, because living alone may also be an important mediating factor that explains some of the negative caregiver outcome, elderly individuals that live alone are also identified as a group at risk of loss of mental health and wellbeing. Because spouses of dementia patients often are old themselves, this study indicates that the ability to sufficiently care for spouses when respective partners with dementia are placed in nursing homes may absolutely require extra effort from the public health care system.

The distress associated with having a partner with dementia in a nursing home versus having such a partner residing at home may indicate that interventions aimed at delaying nursing home admission may be important. This interpretation of the results should be kept open even if we cannot conclude that the trend of the worst mental health in caregivers with a spouse in a nursing home is due to the nursing home transmission or to the progressing state of dementia. A delay of nursing home admission is probably important both for the caregiver and certainly because of the high costs of institutional care for society. In supporting the caregiver, one must focus on community settings that can provide necessary help at home, enabling caregivers to continue to live with and provide care for their respective partners in the home for as long as possible.

Although our study did not demonstrate moderator effects, it did indicate that factors that add to vulnerability to mental distress in the general population also account for such vulnerability in caregivers. However, the negative results regarding moderator effects might well be due to limited statistical power. Larger and longitudinal studies are needed to identify factors that predispose caregivers to greater emotional resilience or vulnerability.

## Competing interests

The authors declare that they have no competing interests.

## Authors’ contributions

HA performed the statistical analyses and drafted the manuscript. All authors contributed to the study’s design, preparation of the data, interpretation of the analyses, and critical revision of the manuscript. All authors read and approved the final manuscript.

## Pre-publication history

The pre-publication history for this paper can be accessed here:

http://www.biomedcentral.com/1471-2458/14/413/prepub
